# A Rare Case of Methyldopa-Induced Hepatitis

**DOI:** 10.7759/cureus.84873

**Published:** 2025-05-27

**Authors:** Bruno Bonito, Joana Cartucho, Monica F Silva, Inês Ferreira Maia, Maria do Rosário Ginga

**Affiliations:** 1 Internal Medicine, Centro Hospitalar Barreiro Montijo, Barreiro, PRT

**Keywords:** dili, hepatitis, jaundice, liver failure, methyldopa

## Abstract

Methyldopa is a centrally acting antihypertensive agent commonly used in the management of hypertension. It has been associated with rare but serious hepatotoxic effects. Methyldopa-induced hepatitis is an uncommon but potentially devastating adverse effect that can mimic autoimmune hepatitis (AIH) both clinically and histologically. This report presents the case of a 31-year-old woman with a history of hypertension who developed acute hepatitis following the initiation of methyldopa. The patient presented with fatigue, jaundice, and elevated liver enzymes approximately six weeks after starting therapy. Viral, autoimmune, and metabolic causes of hepatitis were excluded through comprehensive testing. Liver function normalized following the discontinuation of methyldopa, and the patient recovered fully, supporting a diagnosis of drug-induced liver injury (DILI). Causality was assessed using the Roussel Uclaf Causality Assessment Method (RUCAM), yielding a score of 9, indicating a 'highly probable' link between methyldopa and the observed hepatitis.

## Introduction

Drug-induced liver injury is an uncommon but important cause of acute liver failure [1]. Its clinical presentation and histopathological features often mimic other hepatic disorders, particularly autoimmune hepatitis, making the diagnosis especially challenging and frequently one of exclusion [1,2].

Multiple drugs are known to cause acute and chronic liver failure with different mechanisms involved [2]. Methyldopa, an alpha-2 adrenergic agonist, is commonly used to manage hypertension, particularly in pregnant women, due to its favorable safety profile [3]. While generally well-tolerated, methyldopa has been associated with hepatotoxicity, presenting either as acute or chronic hepatitis. Although methyldopa-induced hepatitis is considered rare, the precise incidence remains uncertain, and the estimated incidence is thought to range from 0.1% to 0.5% [4].

The exact pathophysiology of methyldopa-induced liver injury remains incompletely understood but is believed to involve both immune-mediated and toxic mechanisms. Immune-mediated injury likely results from the formation of reactive drug metabolites that bind to liver proteins, creating neoantigens that trigger a T-cell-mediated autoimmune response. This process is supported by findings of autoantibodies and histological patterns resembling autoimmune hepatitis in affected patients [1,2,5]. In contrast, toxic injury may occur via direct hepatocyte damage due to oxidative stress or mitochondrial dysfunction induced by the drug or its metabolites, although this mechanism is considered less prominent in methyldopa-related cases [2,4].

## Case presentation

We present the case of a 31-year-old, Caucasian woman with a past medical history significant for class III obesity (BMI ≥ 40 kg/m²) and asthma, who presented with a one-week history of right upper quadrant abdominal pain, described as dull and persistent, radiating to the back, with no relief factors. She also reported malaise, fatigue, nausea, and jaundice.

Associated symptoms, such as weight loss, diarrhea, fever, anorexia, or vomiting, were also denied. There was no recent history suggestive of viral or bacterial infection.

The patient had recently been diagnosed with hypertension and was started on alpha-methyldopa at a dose of 750 mg per day, six weeks prior to hospital admission. She denied alcohol consumption, paracetamol overdose, recreational drug use, ingestion of wild mushrooms, herbal supplements, or recent travel. There was no preceding viral prodrome. She lived in an urban setting, had no tattoos or body piercings, and denied tobacco use. There was no history of blood transfusion, high-risk sexual behavior, or known exposure to sexually transmitted infections.

In addition to methyldopa, her regular medications included a budesonide and formoterol inhaler for asthma. She denied any known drug or food allergies.

There was no known personal or family history of liver disease, autoimmune disorders, metabolic conditions, or other chronic illnesses known to affect hepatic function.

Physical examination revealed mild jaundice and tenderness on palpation of the right upper quadrant. There was no evidence of ascites, hepatosplenomegaly, or palpable lymphadenopathy. After anti-hypertensive treatment, her blood pressure remained within the normal range, with no history of hypotensive episodes or cardiovascular events.

**Figure 1:**
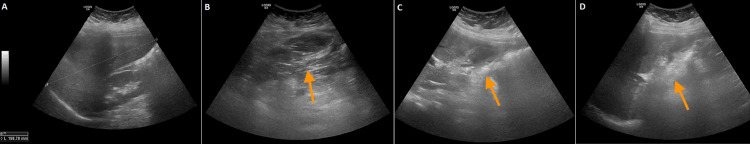
Abdominal ultrasound showing hepatomegaly with mild hepatic steatosis (A) and gallbladder (orange arrows) with sludge, without wall thickening (B, C, D)

Abdominal ultrasound demonstrated hepatomegaly with mild steatosis. The gallbladder contained sludge but showed no wall thickening, and there was no biliary ductal dilatation. No focal hepatic lesions or ascites were reported (Figure 1).

All relevant laboratory findings, including complete blood count, renal function, serum electrolytes, liver function tests, coagulation profile, inflammatory markers, viral and infectious serologies, and an extensive autoimmune workup, have been comprehensively summarized in Table 1.

**Table 1:** Laboratory investigations INR: international normalized ratio; HSV: herpes simplex virus; EBV: Ebstein-Barr virus; CMV: cytomegalovirus; HIV: human immunodeficiency virus; ANA: antinuclear antibody; anti-DNA: anti-double-stranded DNA; anti-SSA: anti-Ro protein antibodies, anti-SSB: anti-La protein antibodies; anti-Sm: anti-Smith; anti-RNP: anti-ribonucleoprotein antibodies; anti-LKM: anti-liver-kidney microsomal antibodies; p-ANCA: perinuclear anti-neutrophil cytoplasmic antibodies; anti-gp210: anti-gp210 protein antibodies; anti-LC1: anti-liver cytosolic antigen type 1; anti-SLA/LP: anti-soluble liver antigen/liver pancreas antibodies; anti-Sp100: anti-Sp100 protein antibodies; ASMA: anti-smooth muscle antibodies

Test	Result	Reference range
Hemoglobin (g/dL)	13.8	12.0-15.0
Leukocytes (x10^9^/L)	5.60	4-10
Platelets (x10^9^/L)	188	150-400
Urea (mg/dL)	16	10-50
Creatinine (mg/dL)	0.68	0.55-1.02
Sodium (mmol/L)	139	136-145
Potassium (mmol/L)	3.6	3.5-5.1
Cloride (mmol/L)	107	98-107
Total bilirrubin (mg/dL)	5.62	< 1.2
Direct bilrrubin (mg/dL)	4.12	0.1-0.5
Aspartate aminotransferase (UI/L)	1369	< 34
Alanine aminotransferase (UI/L)	2859	< 55
Alkaline phosphatase (UI/L)	110	< 150
INR	1.35	0.8-1.2
Lipase (UI/L)	34	8-78
Amylase (UI/L)	39	25-125
C-reactive protein (mg/L)	13	< 5.0
Hepatitis A, B, C, and E	Negative	
Antibodies for toxoplasmosis, HSV, EBV, and CMV	Negative	
HIV I/II	Negative	
Huddlesson reaction	Negative	
Rose Bengal agglutination test	Negative	
Widal test	Negative	
Weil-Felix test	Negative	
ANA	Negative	
Anti-DNA (UI/mL)	Negative	< 100
Anti-SSA, anti-SSB, anti-Sm, anti-RNP (RU/mL)	Negative	< 18
anti-LKM (U/mL)	Negative	< 18
p-ANCA (UA/mL)	Negative	< 20
anti-gp210, anti-LC1, anti-SLA/LP, anti-Sp100	Negative	
ASMA	Negative	
Lupus anticoagulant	Negative	
Anti-cardiolipin antibodies (U/mL)	Negative	< 11
Anti-β2 Glycoprotein I antibodies	Negative	< 18
Ferritin (ng/mL)	3876	30-204
Transferrin saturation (%)	32	15-50
Ceruloplasmin (mg/dL)	28	20-50
24-hour urinary copper excretion (mcg/24h)	12	10-30

The markedly elevated ferritin level observed in our patient was consistent with an acute-phase inflammatory response rather than iron overload. Ferritin acts as an acute-phase reactant and can be significantly increased in systemic inflammation, infection, or liver injury. Normal transferrin saturation and negative iron studies effectively exclude hereditary or acquired iron overload syndromes such as hemochromatosis. Furthermore, Wilson’s disease was excluded by normal ceruloplasmin levels and 24-hour urinary copper excretion, ruling out this differential diagnosis.

**Figure 2:**
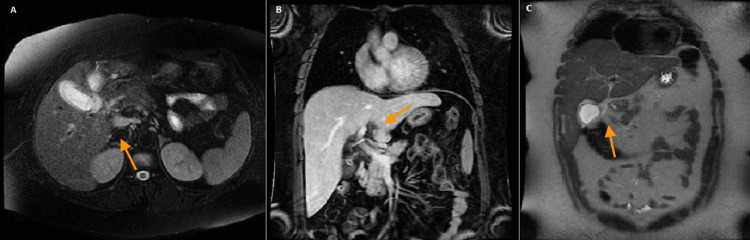
MRCP axial (A) and coronal (B, C) sequences showing mild hepatomegaly and lymph nodes at the hepatic hilum (orange arrows) MRCP: magnetic resonance cholangiopancreatography

Magnetic resonance cholangiopancreatography (MRCP) revealed mild hepatomegaly, with the right hepatic lobe measuring 20 cm in longitudinal diameter and the left lobe 9.8 cm anteroposteriorly. Hepatic morphology did not suggest chronic liver disease, though relative hypertrophy of the caudate lobe was noted. There was mild diffuse hepatic steatosis, without evidence of focal lesions or biliary ductal dilatation, either intrahepatic or extrahepatic. The gallbladder demonstrated mild wall thickening, without gallstones. No features of cholecystitis were present. Three lymph nodes were identified at the hepatic hilum, the largest measuring 33 x 16 mm in the interportocaval region. These findings were suggestive of a probable hepatic inflammatory process. No lymphadenopathy was seen in other regions, aside from a few minimally prominent subcentimetric nodes in the para-aortic area, deemed of low significance. The pancreas and Wirsung duct were normal. The spleen, adrenal glands, and kidneys appeared unremarkable. There was no ascites (Figure 2).

Considering the clinical context of acute hepatitis and systemic inflammation, the lymphadenopathies are most consistent with a reactive process secondary to hepatic inflammation. There was no radiologic evidence suggestive of pathological lymphadenopathy such as malignancy or granulomatous disease. Given the temporal association between methyldopa initiation and the onset of liver failure, along with the exclusion of infectious, autoimmune, metabolic, and obstructive causes, and the subsequent clinical and biochemical improvement following drug discontinuation, a diagnosis of methyldopa-induced hepatitis was established. Although a liver biopsy can help distinguish drug-induced liver injury from autoimmune hepatitis, it was not pursued in this case. The decision was based on the patient’s prompt clinical and biochemical improvement, the absence of autoimmune markers, and the potential risks associated with liver biopsy. Additionally, the patient’s obesity was considered a relevant factor that could increase the technical difficulty and complication risk of the procedure.

Methyldopa was discontinued, and the patient was transitioned to candesartan, an angiotensin receptor blocker, for blood pressure management. Systemic corticosteroids, which are sometimes considered in cases of autoimmune-like drug-induced liver injury, were not administered, as the patient demonstrated early signs of clinical and biochemical improvement, suggesting a self-limited drug-induced liver injury. The patient’s inhaled asthma therapy (budesonide/formoterol) was not administered during hospitalization, as the patient's asthma was controlled at the time. Over the subsequent six weeks, liver enzymes and bilirubin levels gradually normalized, and the patient achieved full recovery (Table 2).

**Table 2:** Evolution of liver function tests and INR during hospitalization and follow-up AST: aspartate aminotransferase; ALT: alanine aminotransferase; ALP: alkaline phosphatase; GGT: gama-glutamyl transpeptidase; INR: international normalized ratio

	Admission	After 5 days	After 10 days (discharge)	After 6 weeks
AST UI/L (ref < 34)	1369	1227	655	26
ALT UI/L (ref < 55)	2859	2272	1321	45
ALP UI/L (ref <150)	110	102	89	56
GGT UI/L (ref 9-36)	214	196	132	58
Bilirubin mg/dL (ref <1.2)	5.62	11.3	3.33	1.03
INR (ref 0.8-1.2)	1.35	1.24	1.11	1.01

To further support the diagnosis of drug-induced liver injury (DILI), we applied the Roussel Uclaf Causality Assessment Method (RUCAM). The patient achieved a score of 9 points, indicating a highly probable causal relationship between methyldopa and the hepatic injury. This score was based on the temporal relationship between drug initiation and symptom onset, rapid improvement upon drug discontinuation, exclusion of alternative causes, and absence of re-exposure. The use of this standardized tool reinforces the strength of the causal association in this case.

## Discussion

We present the case of a female patient with abdominal pain, malaise, jaundice, and marked transaminase elevation who was diagnosed with acute hepatitis following the recent initiation of methyldopa for hypertension. The diagnosis of methyldopa-induced hepatitis was obtained after the exclusion of other causes of acute hepatitis and was strengthened following significant clinical and biochemical improvement once methyldopa was discontinued.

Methyldopa has been used for decades as a first-line antihypertensive, particularly in pregnant women, due to its safety profile in gestation [3]. Nonetheless, methyldopa-induced liver injury, although rare, remains clinically significant due to its potential for severe outcomes, including acute and chronic hepatic failure, potentially resulting in permanent hepatic damage. The pathogenesis is not entirely understood. Hepatic injury related to methyldopa is considered idiosyncratic and immune-mediated. While often reversible, it can lead to significant morbidity if not promptly recognized [1,2]. The onset typically occurs within 2 to 12 weeks of initiating therapy, aligning with our patient's presentation six weeks after starting methyldopa. Clinical manifestations can range from asymptomatic elevations in liver enzymes to symptomatic hepatitis with jaundice, as observed in our case [3,4].

This case aligns with features of drug-induced liver injury and specifically fits the phenotype of autoimmune-like drug-induced liver injury, which can resemble idiopathic autoimmune hepatitis (AIH) in clinical presentation. However, important distinctions exist. Autoimmune-like DILI typically lacks sustained autoantibody positivity and does not require long-term immunosuppression, in contrast to classical AIH [1,2]. The patient's comprehensive evaluation ruled out other causes of hepatitis, including viral infections and autoimmune diseases. Imaging studies revealed hepatic steatosis and inflammatory changes without biliary obstruction. The temporal relationship between methyldopa initiation and the onset of symptoms, coupled with the exclusion of other etiologies and the patient's improvement upon discontinuation of the drug, are features that are more consistent with autoimmune-like DILI than idiopathic AIH [5,6], which supports the diagnosis of methyldopa-induced DILI. Notably, some methyldopa-induced hepatotoxicity cases have demonstrated histological overlap with autoimmune hepatitis, including plasma cell infiltration and interface hepatitis [1,6-8]. Histological confirmation via liver biopsy is often considered in ambiguous cases; however, in this patient, the non-invasive workup and clinical course were sufficiently conclusive.

Unlike autoimmune hepatitis, drug-induced hepatitis often resolves with drug discontinuation alone. Corticosteroids may be considered in severe or persistent cases, particularly when autoimmune features are present [7]. While the literature is not consensual, in previously reported cases of drug-induced autoimmune-like hepatitis, corticosteroids have typically been administered in doses comparable to those used in idiopathic autoimmune hepatitis, with initial oral prednisone doses of 30-60 mg/day or equivalent [1,5].

In our patient, clinical and transaminase improvement after drug discontinuation and supportive treatment validates the diagnosis of a transient toxic or immune-mediated insult rather than a persistent autoimmune process, and therefore, the patient was not considered for corticotherapy. Although methyldopa is generally considered safe, hepatotoxicity has been reported in various populations, including those with no identifiable risk factors [3,4]. Our patient’s class III obesity raises the question of whether underlying metabolic stress may have contributed to hepatic vulnerability. Obesity is associated with hepatic steatosis and low-grade inflammation, both of which may predispose individuals to DILI by altering drug metabolism and increasing oxidative stress. While current literature does not establish a direct link between obesity and methyldopa-induced hepatitis, this case suggests that metabolic risk factors could influence susceptibility and should be considered when evaluating hepatotoxic risk.

This case underscores the importance of a detailed medication history and a broad differential when evaluating unexplained hepatitis. Clinicians must remain vigilant for uncommon but serious adverse effects of commonly used medications such as methyldopa, particularly in populations where it remains a first-line antihypertensive, like pregnant and/or young women. Early recognition and prompt discontinuation of the offending drug are vital to prevent progression to severe liver injury.

## Conclusions

Methyldopa-induced hepatitis, while rare, should be considered in the differential diagnosis of acute hepatitis, especially in patients who were recently started on this drug. Key diagnostic features include a clear temporal relationship between drug exposure and symptom onset, resolution of symptoms upon drug withdrawal, and the absence of autoimmune serologic markers. In contrast to autoimmune hepatitis, drug-induced liver injury typically demonstrates spontaneous improvement without immunosuppressive therapy and does not relapse after recovery.

A thorough clinical history, exclusion of alternative etiologies, and a clear temporal association with medication use are crucial for diagnosis. Prompt discontinuation of the offending agent often leads to complete recovery, avoiding unnecessary treatment and invasive diagnostic procedures. Clinician awareness and early recognition are key to preventing progression to liver failure and optimizing patient outcomes.
